# Development of Novel S-Protective Thiolated-Based Mucoadhesive Tablets for Repaglinide: Pharmacokinetic Study

**DOI:** 10.3390/polym14173529

**Published:** 2022-08-28

**Authors:** Nabil A. Alhakamy, Nimbagal Raghavendra Naveen, Shashank Gorityala, Mallesh Kurakula, Khaled M. Hosny, Awaji Y. Safhi, Deena M. Bukhary, Haitham A. Bukhary, Fahad Y. Sabei, Rayan Y. Mushtaq, Samar S. Murshid

**Affiliations:** 1 Department of Pharmaceutics, Faculty of Pharmacy, King Abdulaziz University, Jeddah 21589, Saudi Arabia; 2 Center of Excellence for Drug Research and Pharmaceutical Industries, King Abdulaziz University, Jeddah 21589, Saudi Arabia; 3 Department of Pharmaceutics, Sri Adichunchanagiri College of Pharmacy, Adichunchanagiri University, B.G. Nagar 571448, India; 4 Bioanalytical Chemistry, Labcorp Drug Development, Madison, WI 53704, USA; 5 Product Development Department, CURE Pharmaceutical, Oxnard, CA 93033, USA; 6 Department of Pharmaceutics, College of Pharmacy, Jazan University, Jazan 45142, Saudi Arabia; 7 Department of Pharmaceutics, College of Pharmacy, Umm Al-Qura University, Mecca 24382, Saudi Arabia; 8 Department of Pharmaceutics, College of Clinical Pharmacy, Immam Abdulrahman Bin Faisal University, Dammam 34212, Saudi Arabia; 9 Department of Natural Products and Alternative Medicine, Faculty of Pharmacy, King Abdulaziz University, Jeddah 21589, Saudi Arabia

**Keywords:** repaglinide, thiolation, mucoadhesion, xanthan gum, tablet

## Abstract

Mucoadhesive polymers have an essential role in drug localization and target-specific actions in oral delivery systems. The current work aims to develop and characterize a new mucoadhesive polysaccharide polymer (thiolated xanthan gum-TXG and S-Protected thiolated xanthan gum-STX) that was further utilized for the preparation of repaglinide mucoadhesive tablets. The thiolation of xanthan gum was carried out by ester formation through the reaction of the hydroxyl group of xanthan gum and the carboxyl group of thioglycolic acid. Synthesis of TXG was optimized using central composite design, and TXG prepared using 5.303 moles/L of TGA and 6.075 g/L of xanthan gum can accomplish the prerequisites of the optimized formulation. Consequently, TXG was further combined with aromatic 2-mercapto-nicotinic acid to synthesize STX. TXG and STX were further studied for Fourier-transform infrared spectroscopy, rheological investigations, and Ellman’s assay (to quantify the number of thiol/disulfide groups). A substantial rise in the viscosity of STX might be due to increased interactions of macromolecules liable for improving the mucosal adhesion strength of thiolated gum. STX was proven safe with the support of cytotoxic study data. Mucoadhesive formulations of repaglinide-containing STX showed the highest ex vivo mucoadhesion strength (12.78 g-RSX-1 and 17.57 g- RSX-2) and residence time (>16 h). The improved cross-linkage and cohesive nature of the matrix in the thiolated and S-protected thiolated formulations was responsible for the controlled release of repaglinide over 16 h. The pharmacokinetic study revealed the greater AUC (area under the curve) and long half-life with the RSX-2 formulation, confirming that formulations based on S-protected thiomers can be favorable drug systems for enhancing the bioavailability of low-solubility drugs.

## 1. Introduction

Mucoadhesion is a process that describes the interactivity of polymer and the mucus layer. This interaction forms strong adhesion bonds through electrostatic, cross-linkage, wetting action, adsorption, and chemical bonds [1,2]. Mucoadhesive systems are formulated to deliver drugs at specific anatomical sites such as the nose [3], buccal cavity [4,5], gastrointestinal tract [6], rectum [7], vagina etc. [8,9]. Single or multiple adhesive polymers can be employed to render adequate mucoadhesive properties to the drug system [10]. To improve mucoadhesion ability, polymer composites and chemical alterations of polymers can be carried out [11].

Thiolation of the mucoadhesive polymers renders the potential to make disulfide bonds (inter-/intrachain) within the polymeric system and can significantly enhance their cohesive nature. The chemical reaction of thiol moiety with mucin-containing cysteine results in the development of strong covalent bonds [12,13]. Thiomers, in contrast to the unaltered polymers, exhibit good adhesive properties that are adequate to restrain the drug at required target sites for a longer duration. In addition, thiolated polymers have the effects of enzyme inhibition, improved penetration, controlled release, and thermal stability [14]. The thiolation process was successfully applied to improve the mucoadhesive properties of different gums such as tragacanth [15], moringa gum [16], xanthan gum [17], gellan gum [18], tamarind gum [19,20,21], and psyllium husk [22]. However, the oxidation of thiol moiety modifies their interaction with mucus glycoproteins containing cysteine, thus restricting their mucoadhesive potency [23,24]. Thiolated polymers were S-protected with 2-mercapto-nicotinic acid (MA), further enhancing thiol groups’ reactivity due to electron shrinkage in the pyridine π system [25,26].

Using natural gums and mucilage of plants is impervious as a pharmaceutical excipient, particularly in the formulation of controlled drug forms [27]. These substances’ physical and chemical characteristics can easily be modified to achieve the requirements of an ideal drug delivery system [28]. Xanthan gum is a natural, high-MW polysaccharide obtained by the fermentation of sugars with Xanthomonas campestris bacteria (usually present on the leaves of green veggies, especially in the cabbage family) [29]. It was studied extensively for different pharma, cosmetics, and food applications as an excipient, stabilizing agent, viscosity enhancer, hardening agent, and emulsifying and suspending agent.

Alterations of xanthan gum have been carried out in previous studies through grafting reactions using acrylamide that revealed an improvement in hydration properties to the original polysaccharide, grafting reaction with ethyl acrylate employed in the area of separation and purification as they are differentiated remarkably by greater absorptivity, renewability, and stability [30,31]. Chemical alteration with methanal and carboxymethylation was observed to enhance the drug dissolution, proving the mucoadhesive features of the polymers [32]. To the best of our knowledge, S-protected thiolated xanthan gum (STX), and its mucoadhesion potency, was not described in the literature.

Repaglinide was chosen as a drug model in this work to evaluate the potential of STX in developing a mucoadhesive delivery system. Repaglinide has distinct anti-diabetic action. It binds to pancreatic β cells, stimulates insulin, and impedes ATP-dependent potassium channels. Nevertheless, drug usage is restricted because of its short biological half-life (60 min) and only 50% bioavailability [33]. In addition, repaglinide induces hypoglycemia when taken orally and causes side effects such as musculoskeletal pain, headache, and gastrointestinal effects [34]. Repaglinide is an appropriate target for formulating gastro-retentive forms because of its short activity, quick clearance, enzyme stability, and stomach absorption window. The current work describes the application of STX in developing gastro-retentive, mucoadhesive dosage forms of repaglinide.

## 2. Materials and Methods

### 2.1. Materials

Aurobindo Pharma, Hyderabad, India presented the repaglinide. Xanthan gum was procured from Loba Chemie Pvt Ltd.; Mumbai, India. Thioglycolic acid (TGA), MA, and hydrochloric acid were obtained from SD Fine-Chem Ltd. (Mumbai, India). Other solvents and chemicals used were of analytical grade.

### 2.2. Thiolation of Xanthan Gum [TXG]

Thiolation of xanthan gum was carried out through esterification using thioglycolic acid under the influence of catalytic HCl, as reported by Meenakshi Bhatia et al. [35]. This process was conducted using 2–6 moles/L of TGA for each mole of hydroxyl (-OH) group in xanthan gum. An amount of 4–8 g/L of Xanthan gum was allowed to dissolve in 250 mL of hot water along with TGA (4.2 mL) and HCl (7 N). The mixture was subjected to a reflux reaction at 80 °C for two and half hours. The reaction mixture was allowed to cool and further precipitated with methyl alcohol. The resultant precipitates were cleaned twice using methanol and kept in the oven at 50 °C for drying.

#### 2.2.1. Experimental Design

Preparation of TXG is standardized by the statistical model RSM. The concentrations of TGA (X_1_) and xanthan gum (X_2_) were chosen as individual parameters at five distinct levels, encoded as −1.414, (low), −1, 0 (medium), +1 and +1 (high). These parameters were scrutinized for their influence on viscosity and mucoadhesion strength [MS] using Design Expert Version 12 (Stat Ease Inc., USA), originating from 13 experiment runs [36,37]. Table 1 represents the total experiment plan, coded and actual values of chosen parameters, and restraints of the chosen responses. Analysis of variance (ANOVA) was used to validate the developed polynomial equations. In addition, various statistical tools were employed in all test runs to select the best-fit model. A quadratic design was employed in every test run to quantify the outcome response and regression analysis.

(1) $$\begin{array}{ll} Y_{i\left(Quadratic\right)}= & b_{0}+b_{1}X_{1}+b_{2}X_{2}+b_{3}X_{3}+b_{4}X_{1}X_{2}+b_{5}X_{1}X_{3}\\ & +b_{6}X_{2}X_{3}+b_{7}X_{1}^{2}+b_{8}X_{2}^{2}+b_{9}X_{3}^{2} \end{array}$$

where

*Y_i_*—Chosen response or dependent variable,

*b*_0_—Computed response

*b_i_*—The estimated coefficient for main effects (X_1_, X_2_, X_3_); interaction terms of main effects (X_1_X_2_, X_2_X_3_, X_1_X_3_); and polynomial terms of independent variables (X_1_^2^, X_2_^2^, X_3_^2^) 

#### 2.2.2. Rheological Test

The viscoelastic nature of every compound was determined using a plate–plate combined rheometer (RotoVisco RT20, Haake GmbH, Karlsruhe, Germany). In summary, firstly, 0.01 g of polymer sample was hydrated in deionized water and then diluted with PBS (pH 7.4) to achieve a solution of 0.5% (*m/v*). Following the buffer and an equilibration time in an incubator for 3 or 24 h at 37 °C, 500 μL of each polymer solution was placed in the viscometer. As mentioned earlier, the apparent viscosity (η) was determined for all the samples. The tangential stress was set to around 0.5−500 Pa, and the two plates were kept apart at a 0.5 mm distance and maintained at 37 °C [38]. 

#### 2.2.3. Mucoadhesion Studies via Rotating Cylinder

An amount of 200 mg of prepared polymers was compressed using a 13 mm-diameter die on an infra-red hydraulic press (Perkin Elmer, England) using a compression force of 5 tons for 30 s. The prepared disks had a 13 mm diameter and 1.26 mm average thickness. The rotating cylinder established the time of adhesion of compressed discs to the goat gastric mucosa. Discs were attached to the freshly excised porcine intestinal mucosa (approx. 12 cm^2^) before being spanned onto a stainless-steel cylinder (diameter 2.5 cm, height 3.7 cm; apparatus I Basket, USP). After that, the cylinder was displaced into the dissolution apparatus (USP Type-I) and completely immersed in the 100 mL phosphate buffer solution of pH 6.8 at 37 ± 0.2 °C. The cylinder was rotated at a speed of 125 rpm. Every 30 min, the changes in the test system were perceived visually and registered until all discs were either disintegrated or detached from the mucosa. This test was performed at least five times for each polymer disc.

### 2.3. Synthesis of STX

**Figure 1:**
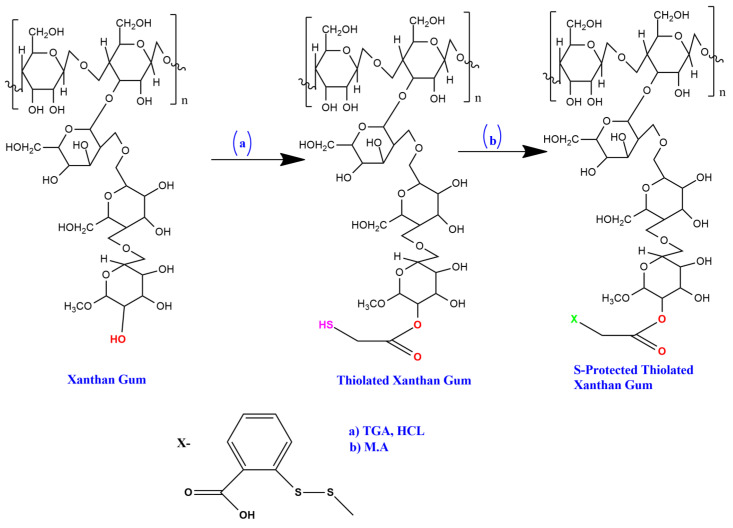
Schematic representation of synthesis of TXG and STX. (**a**) Synthesis of TXG using TGA and (**b**) synthesis of STX using M.A.

Xanthan gum consists of pentasaccharide repeat units, comprising glucose, mannose, and glucuronic acid in the molar ratio 2:2:1. To prepare STX, thiolated xanthan gum was combined with aromatic MA (dimeric form) through a thiol–disulfide exchange reaction [38,39]. In brief, 0.4 g of thiolated xanthan gum was dismissed in 15 mL of water, and, further, dimeric MA of 100 mg was mixed with the solution by constant stirring, and pH of 8.0 was maintained by adding 1 M sodium hydroxide. The mixture was agitated for four hours at ambient conditions without light. The resultant mix was subjected to dialysis through a cellulose membrane (MWCO 100–500 Da) dialyzing tube, Spectra/Por. The resultant solution allowed freeze drying to achieve the protected product (STX). The final STX was preserved at 4 °C until further use [Figure 1].

### 2.4. Characterization of Xanthan Gum, TXG and STX

#### 2.4.1. Fourier-Transform Infrared Spectroscopy

The FTIR spectrum was recorded at room conditions with an IR spectrophotometer (Perkin Elmer Instruments, North Billerica, MA, USA). Qualitative assessment was carried out and readings were noted to evaluate peak fashions and comparison studies [22]. The FTIR spectra of xanthan gum, TXG, and STX were recorded using KBr disc and analyzed at 400–4000 cm^−1^.

#### 2.4.2. Rheological Test

The viscosity of xanthan gum, TXG and STX was evaluated as per the procedure mentioned in Section 2.2.2 [40]. 

#### 2.4.3. Quantitative Assay of Thiol/Disulfide/MNA Groups

The number of thiol and disulfide groups of STX was estimated through the quantitative study by partial and total Ellman’s assay, respectively [41]. An amount of 0.5 mg of samples was dissolved in 500 μL of PBS (0.5 M at pH 8) to determine MA linked to polymer. A fresh reduced glutathione solution was utilized (10 mg/5 mL) to release MA, and the resultant mixture was incubated for up to 2 h at 37 °C. The free MA generated through thiol–disulfide exchange reaction with glutathione was analyzed, and absorbance was measured at 354 nm using Perkin Elmer VICTOR X3 Multilabel Plate Reader. A control without glutathione was used to determine the absorbance of unbounded MA. TO was used for comparison purposes using the method mentioned above.

### 2.5. Formulation of Mucoadhesive Tablets of Repaglinide

The gastro-retentive mucoadhesion tablets of repaglinide were formulated with 8% polyvinylpyrrolidone (PVP) K30 in 80% ethyl alcohol as a granulation medium. The required quantity of repaglinide, TXG, STX, and all other excipients (Table 2) were admixed rigorously and allowed to pass through #80 mesh. The desired amount of the granulating medium was mixed into the powder blend and sieved through #10–12 to obtain wet granules. The obtained granules were kept for drying at 55–60 °C for about 1 h, and moisture was kept around 3–5%. The dried granules reserved on #14–20 mesh were lubricated using a specified quantity of magnesium stearate and talc. Eventually, the tablets were prepared by the compression method in the Rotary tablet press machine (Chamunda pilot press, Ahmedabad, Gujarat, India) with flat-faced 8 mm punches, and the compressive force was tuned accordingly to achieve a tablet hardness of 8–9 kg/cm^2^.

### 2.6. Evaluation Tests

#### 2.6.1. Quality Control Tests

The prepared tablets were analyzed for thickness, weight variation, friability, and hardness [42]. A thickness test was carried out using an automated screw gauge, and a Monsanto tester assessed tablet hardness. Uniformity in weight was checked through electronic analytical balance (Citizen scales Pvt. Ltd., Coimbatore, India), and friability test was carried out in Roche friabilator (Electrolab-EF-2). In addition, diametric fracture (DF) was inspected, and CSFR (crushing strength/friability ratio) was calculated depending on hardness and friability values. All the prepared tablets’ weights were noted and then they were grounded into powder using mortar and pestle. The quantity of powder equal to an average tablet mass was weighed and shifted to a flask consisting of 0.1 N HCl buffer solution. The flask was agitated continuously for 2–3 h, and, later, filtration was carried out using Whatman filter paper. Dilutions were made, and drug content was analyzed with a double-beam UV-visible spectrophotometer at 223 nm (AU 2701, Systronics, Mumbai, India).

#### 2.6.2. Swelling Study

The initial weight (W_0_) of tablets of all batches was noted and kept in 100 mL of 0.1 N HCl. After 8 h, tablets were pat-dried, and the final weight (W_t_) was taken [30]. The rate of swelling was measured as percentage gain in the weight.

(2) $$\%\;\mathrm{Swelling}=\frac{W_{t}-W_{0}}{W_{0}}\times 100$$

#### 2.6.3. Ex Vivo Mucoadhesion Time 

Individual gastric mucoadhesive tablets were wetted with buffer (0.1N Hcl), then adhered to the fresh goat stomach mucosa (that was affixed to glass slide through cyanoacrylate) through the application of mild force with fingers for around 25–30 s, and examined for adhesion time by keeping the slide in a beaker, which consisted of 200 mL buffer (0.1N Hcl) at 37 ± 0.5 °C [40]. A stirring speed of 50 rpm was maintained to stimulate the gastric conditions. Tablets were held and observed for around 12 h. 

In addition, the formulated tablets’ residence period was analyzed ex vivo by modifying the Type I USP dissolution apparatus (TDT-08L, Electro lab, India) [43]. Freshly obtained stomach mucosa was affixed to the outer basket surface, and the hydrated tablets were secured to it by applying gentle force. Furthermore, the basket was placed in a suitable medium of 0.1N Hcl (500 mL) at a temperature of 37 ±0.5 °C with a speed of 100 rpm. The time required for detachment was noted.

#### 2.6.4. Ex Vivo Study of Mucoadhesion Strength

The mucosal adhesion test of all the formulations was performed using a texture analyzer (TA-XT plus, Stable MicroSystems, UK). The tablet was fixed to the cylinder-shaped probe with double sticky tape. Prior to keeping on the holder point, the gastric tissue of the pig was equilibrated at 37.0 ± 0.5 °C for 15 min. The tablet affixed to the probe was immersed into media for a specified period and advanced to the test. Furthermore, the disc was moved downwards to approach the drenched tissue with a definite force and maintained for a specific time. At a pre-established test speed, the probe was lifted, and the maximum force (Fmax) that was essential to detach the probe with a tablet from the tissue could be estimated through the software (Texture Exponent 32). The forerunner instrument settings were examined with distinct variables such as test speed (0.5 mm/s), contact time (1 min), contact force (1.0 N), and distance (15 mm). In addition, the probe alone was studied without the tablet sample to test the uniformity of the tissue [44].

### 2.7. Biological Studies: Cell Culture and Cell Viability 

#### Cell Cultures and Viability Test by Resazurin Assay

The resazurin (Alamar blue) assay was conducted on CaCO-2 cell cultures as reported earlier [45,46] with similar incubation conditions. The major advantages of the resazurin assay are that it is relatively inexpensive, uses a homogeneous format, and is more sensitive than other assays. Sample solutions of unaltered, thiolated, and S-protected xanthan gum were made individually, and their microparticles were developed using white MEM (1% *m/v*). White MEM and Triton X-100 (1% *m/v*) were used as positive and negative controls.

### 2.8. In Vitro Drug Release Test

The study was performed by in vitro modified basket method (TDT-08L, USP type I, Electro lab, India) to resemble the in vivo adhesion of the drug system. A slice of fresh stomach mucosa was attached to the base and one of the inner sides of the basket. The test was carried out by attaching the hydrated tablet to the mucosa. Aliquots (10 mL) were withdrawn at various time intervals, and the samples were filtered through a 0.45 µm millipore filter, followed by appropriate dilution, and analyzed for drug content by UV spectrophotometer (UV-1800, Shimadzu, Japan) at 223 nm. 

### 2.9. Bioavailability Studies

In vivo studies were conducted after obtaining approval from the Animal Ethical Committee of the Institution of the Clinical Laboratory Center, Beni-suef, Egypt (Approval no. 18/3-02-22). The test was conducted in healthy albino rabbits with 2.2–2.5 kg weight. The optimized formulation and repaglinide solution were determined for t_max_, AUC_t_, AUMC_t_, average residence time, peak plasma concentrations, and half-life. The data were analyzed using PK Solver, a free menu-driven program for MS Excel written in Visual Basic for Applications. Six rabbits were taken for the test and fed with the test drug preparation. A sample of 2 mL blood was drawn from the ear vein before the drug was given (0 h) and at periodic intervals for 22 h of post-administration. After withdrawal, the samples were immediately shifted to test tubes containing heparin. Subsequently, centrifugation was carried out at 5000 rpm for half an hour, and plasma was collected, stored at −20 °C, and analyzed. Chromatographic system:

The Agilent 1100 HPLC system with a UV detector was used. Ez Chrome software was installed for procuring data and assessing the peaks. Hypersil^®^ BDS column 18 (150 mm × 4.6 mm, 5 μm, Thermo Scientific, Pune, India) was employed for chromatographic segregation and validation. Filtration of the solvent and sample was carried out with 0.2 μm and 0.45 μm membranes (Ultipor^®^ N66^®)^, correspondingly. 

Chromatography conditions

Flow speed: 1 mL/min; 

Column: C_18_ (250 × 4.6 mm, 5µm);

Detector wavelength: 245 nm;

Column temperature: 30 °C;

Injection volume: 10 µL;

Operational time: 8 min; 

Mobile phase: 30:70 *v/v* [Phosphate buffer (pH 2.5): Methanol];

Retention time: 5.821 min.

## 3. Results and Discussion

### 3.1. Optimization of Synthesis of TXG

CCD was used to investigate the impact of the selected variables and their interactions resulting in the maximum viscosity and MS. In total, 13 experimental trials were projected and their observed responses are given in Table 3. The viscosity of experimental formulations was identified in the range of 15.5 to 34.5 mPa-s. MS, which determines the polymer’s mucoadhesion strength, ranged between 1.5 and 8.5 h. All the experimental results were analyzed for the selected responses using an f_x_ model and ANOVA. 

The quadratic model was selected for both all the responses on the basis of the sequential sum of squares (Type-I) and fit summary. Model F-value, *p*-value, and R^2^ values were considered for selecting the model. Additionally, the quadratic model has the highest polynomial order with a *p*-value (level of significance) of <0.0001 [Table 4]. 

For viscosity, the predicted R^2^ of 0.8976 is in reasonable agreement with the adjusted R^2^ of 0.9740, i.e., the difference is less than 0.2. Adeq Precision measures the signal-to-noise ratio. A ratio greater than 4 is desirable. The ratio of 29.2120 indicates an adequate signal. This model can be used to navigate the design space. Similar results were observed for MS [0.8351, 0.9593 and 20.2198] [37]. The accuracy of all these selected models was further confirmed by the normal plot of residuals [47]. The prescribed statistical application was not applied for this as the visual inspection graph is acceptable. For all the selected responses, all the studentized residuals were distributed nearer to the straight line confirming that the chosen model can be accepted statistically [48,49]. Supplementary Figure S1 signifies the experimental run against the residuals as a process of recognizing the lurking variables that effect the responses. A scattered trend was detected within the prescribed limit, thus representing a time-coupled variable slink in the background. The reproducibility of the experiments not only ensures accurate results but also guarantees transparency in understanding the methodology, which can be confirmed with the coefficient of variation (CV) value. The required CV value was comparatively lower (3.56% for EE and 8.88% for MS) than the prescribed (CV < 10%), thus ensuring the consistency and precision of the design. An additional parameter, lack of fit, measures the model’s inability to represent the complete data [50]. As evident from the ANOVA data, lack of fit was found to be non-significant (*p* > 0.05) to confirm the fitness of the selected design. ANOVA was performed to study the impact of quantitative effects of selected factors on responses. The obtained data were subjected to multiple regression to yield polynomial equations. The model F-values of 90.79 and 57.64 imply that all the selected models are significant. 

In the case of viscosity, A, B, A^2^ and B^2^ are significant model terms. The experimental design indicated that viscosity was potentially affected by (i) antagonist effect of polynomial terms of A and B with a *p*-value of <0.0001, and (ii) synergistic effect of A and B terms (with a *p* value < 0.0001 and 0.0061, respectively), with A having the highest effects over all the other terms. The experimental design indicated that MS was potentially affected by (i) antagonist effect of polynomial terms of A and B and (ii) synergistic effect of A and B, the A effects being the highest [Table 5]. 

Equations generated for coded factors,

Viscosity = +27.68 + 5.77 A + 0.6036 B − 0.5000 AB − 0.9275 A^2^ − 3.53 B^2^ (3)

MS = +8.36 + 1.36 A + 0.2703 B − 0.2250 AB − 2.53 A^2^ − 1.75 B^2^ (4)

**Figure 2:**
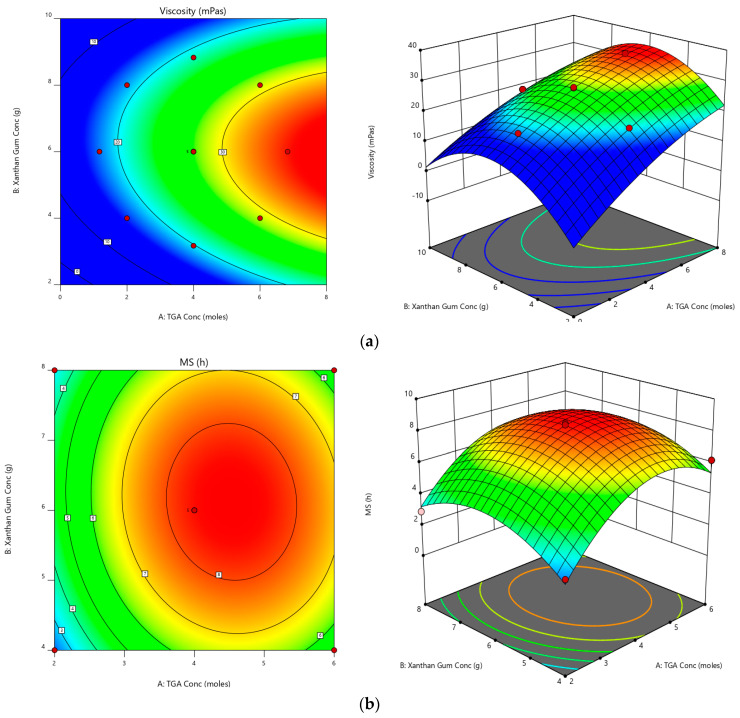
Contour plots and 3-D response surface graphs for (**a**) viscosity and (**b**) MS.

All the above equations can be applied to predict the response for any given concentration of the selected factors. Factor coefficients additionally help in comparing their relative impact on the responses. Contour plots and 3 D RSG (response surface graphs) are crucial to explaining both the interaction and main effect, and the measured responses are pictured with these graphs [Figure 2]. 

**Figure 3:**
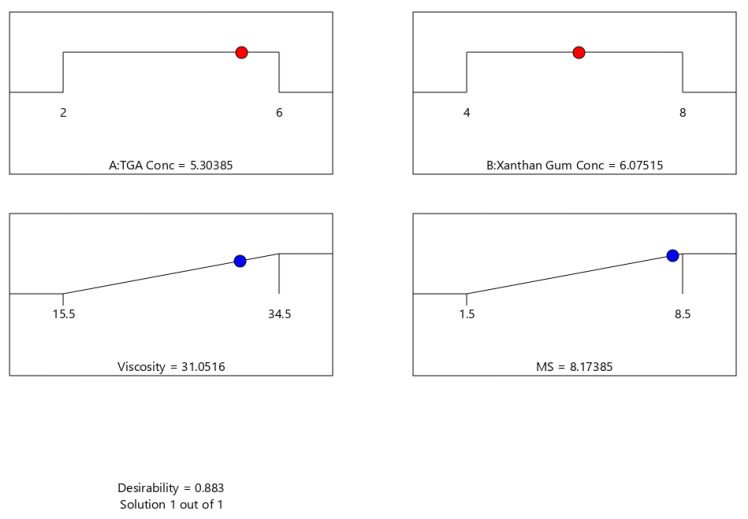
Desirability plot and optimized concentrations to prepare TXG (TGA conc—Moles/L; Xanthan Gum Conc—g/L; Viscosity—mPa-s; MS-h).

Optimizing different series of models obtained from the experimental analysis can be carried out by applying the desirability function [D]. Each response was set to various limits; both the responses were set to maximum to plot the overlay graph [51,52]. All the selected variables were involved in the design space. The combined desirability plot for all the responses showed a maximum D value of 0.883 [Figure 3], which was obtained at optimum concentrations of independent variables, and the critical responses were overlayed in a contour plot. Based on this desirability approach, a TXG prepared using 5.303 moles/L of TGA and 6.075 g/L of xanthan gum can accomplish the prerequisites of the optimized polymer.

Consequently, using these optimized concentrations can result in a viscosity of 31.05, with 8.17 h of MS. By using these projected optimized concentrations, an optimized thiolated xanthan gum (TXG) was prepared and evaluated. The experimental results were compared with theoretical values to validate the experiential design. Relative error was found to be less than 2%, which confirms the preciseness of the design. 

### 3.2. Characterization 

Thiolation of xanthan gum was achieved successfully by the esterification reaction between xanthan gum (hydroxyl group) and TGA (carboxylic group) with a yield of 79 percent. The resultant product had a cream to brown color, with enhanced flow characteristics (the critical repose angle was 32.48 for xanthan gum and 26.94 for TXG, Hausner’s ratio was 1.17 for xanthan gum and 1.05 for TXG) in contrast to the unaltered gum. The FTIR spectrum of XG [Figure 1] exhibited a feature band of absorption at 3523 cm^−1^ because of -OH extension. The peaks observed at 2927 cm^−1^ and 1625 cm^−1^ are attributed to alkanes’ hydroxyl and carboxyl group extensions. The bands at 1420 cm^−1^, 1055 cm^−1,^ and 791 cm^−1^ are due to methyl CH bend, cyclic ether C-O-C extension, and cyclic CH bending. The TXG spectra showed bands of absorption at 3530 cm^−1^ (hydroxyl stretch), 2928 cm^−1^ (methyl CH stretch), and 2567 cm^−1^ (SH stretch of thiol moiety) [Figure 1a]. The peaks observed at 1623 cm^−1^, 1407 cm^−1^, 1054 cm^−1,^ and 789 cm^−1^ have specific features identical to the unaltered polymer. 

**Figure 4:**
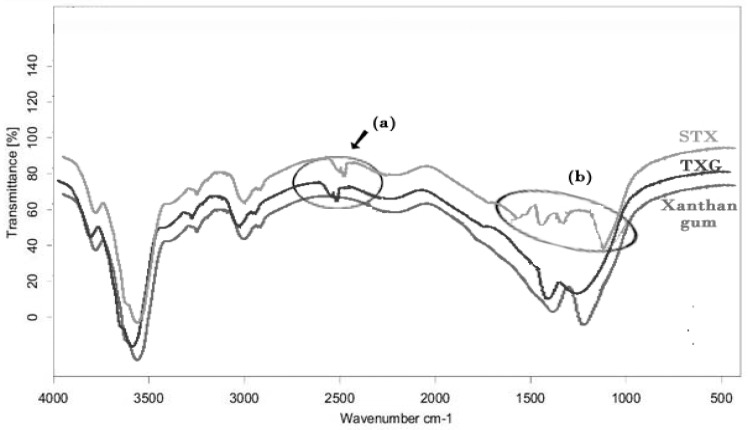
FTIR spectra of xanthan gum, TXG and STX. (**a**) confirms the presence of thiol groups in TXG and STX and (**b**) represents the S-Protected groups of STX.

The protected thiomers benefit from improved thiol stability against broad pH changes and oxygen environment, permitting their usage in microsphere-producing methods. Hence, the STX was designed and formulated to enhance the cohesive nature and stability. Since the TXG gets oxidized in the occurrence of oxygen or solution form, its usage is restricted in formulations of multiparticulate systems. S-protected TXG is coupled with MA, the unbounded thiol moiety, and, therefore, escapes oxidation. The S-protection of thiol moiety was accomplished through the exchange reaction of disulfide and thiol between TXG and MA [53]. FTIR studies confirmed the association of thiomer and the aromatic ligand FT-IR. Figure 4 shows the FTIR spectra of STX. The specific band near 3030 cm^−^^1^ is due to stretch vibrations of aromatic C-H, which was identifiable in the IR spectrum of the STX. At 1166 cm^−^^1^, C-H wagging is observed. In addition, the S-protected thiomer exhibited wide peaks between 1650 and 1450 cm^−^^1^ due to −C=C stretch vibrations, generally four bands of different intensity; this also ensures the occurrence of an aromatic compound [Figure 4b]. 

**Figure 5:**
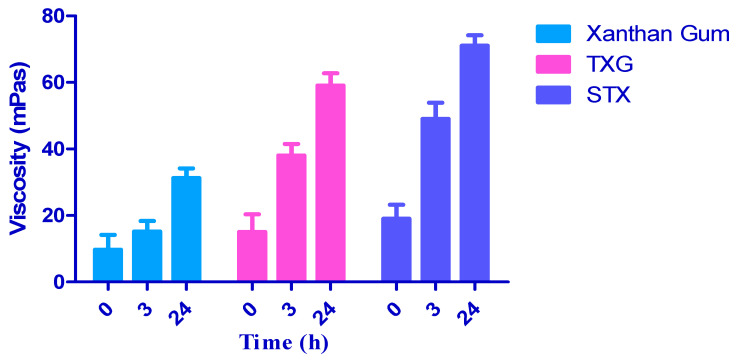
Rheological profile of xanthan gum, TXG, and STX.

Rheological tests were conducted using a plate rheometer to estimate the viscoelastic characters of every polymer sample, and, therefore, interpretations can also be made regarding their cytotoxicities. As a result of adding TGA to xanthan gum, the viscosity of all thiomers improved by a minimum of 1.54-fold (TXG) and 1.96-fold (STX) during 3 h [Figure 5]. Surprisingly, a rise of 2.27× was observed in the viscosity for STX post 24 h of the test, with no significant change in xanthan gum’s viscosity and the S-protected thiomers [54]. At physiological pH, oxidation may occur within the thiolated polymer, resulting in intra-molecular disulfide bond formation after a specified time. Due to this internal cross-linkage, their viscoelastic characters were altered over time. In addition, it could be manifested that the higher the number of thiol moieties disabled on the polymer, the more significant the improvement in viscosity; this finding is correlated with the results of Sakloetsakun et al. [39,55]. 

The Ellman’s assay examined thiolated and S-protected xanthan gum to determine the amount of free and oxidized thiol moieties on the structural back frame, and the findings are shown in Table 6. Ellman’s reagent possesses a disulfide bond that interacts with free thiols (present on the polymer back frame) and forms a yellow product, 2-nitro-5-thiobenzoic acid, that can easily be analyzed at 450 nm. The total quantity of –SH was determined using sodium borohydride to reduce all disulfides computed through Ellman’s reagent. The number of oxidized thiols was calculated by subtracting the free thiol groups from the total amount of thiol groups. TXG exhibited a mean thiol group content of 266.78 ± 13 μmol/g and a mean disulfide group content of 202.29 ± 14 μmol/g. The determination of MA coupled with STX was conducted by adding glutathione to release MA through a thiol–disulfide exchange reaction and subsequently analyzed by photometry. The combined form exhibited an MA content of 219.48 ± 11.8 μmol/g, indicating that around 90 percent of all thiol moieties were S-protected. Hence, S-protection of TXG can render excellent stability against oxidation prior to reaching the mucosal lining.

### 3.3. Formulation of Mucoadhesive Dosage Forms

**Figure 6:**
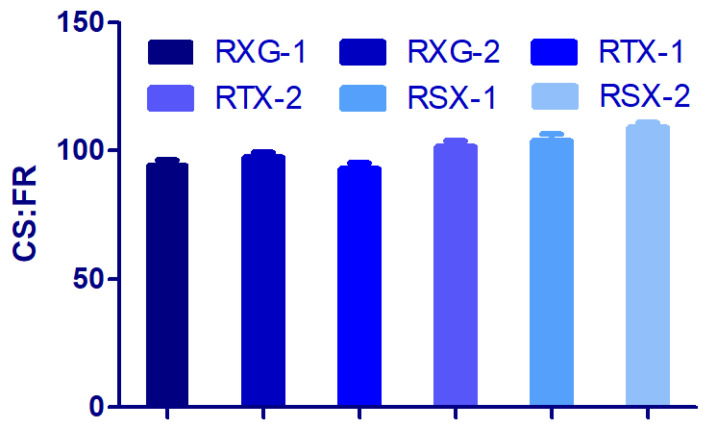
Assessment of the CSFR for prepared batches.

A total of six formulations were planned using the wet granulation method with different concentrations (30 mg and 60 mg) of xanthan gum, TXG, and STX. The formulated granules exhibited good flow characteristics as noticed in the angle of repose (26° to 29°), Hausner’s ratio (1.11–1.18), and compressibility index (9–12%). The compressive force was modified to maintain a crushing strength of 8–9 kg/cm^2^ and passed the DF test. The upper and lower percent of friability for all preparations were found to be 0.084 and 0.1%, with no measurable difference between them. The crushing strength to friability ratio (CSFR) was greater than 80, inferring adequate mechanical tablet strength [Figure 6]. Both weight and thickness variation were kept at <2.5%. The drug content in all the formulations was found to be >95%. 

**Figure 7:**
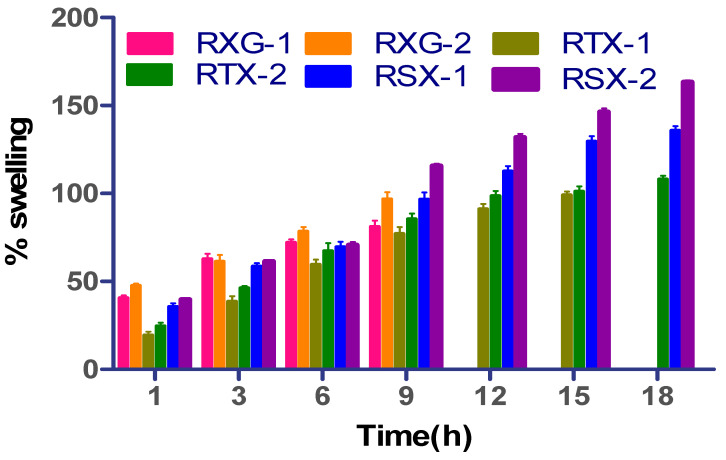
Swelling studies of repaglinide mucoadhesive formulations.

The swelling property of the polymers has a significant effect on cohesive and adhesion nature; thus, water intake capacity was determined for the developed formulation. Figure 6 illustrates the swelling percent of the tablet batches prepared at various periods. The preparations (RXG-1 and RXG-2) containing unaltered xanthan gum swelled fast but were inadequate to sustain the integrity due to the quick hydration and tablet breakdown. The two preparations’ swelling nature by the last 6 h was 72.10 ± 1.89% and 81.24 ±2.58%. The swelling index for RTX-1 and RTX-2 was 91.05 ± 2.13 and 101.78 ±3.06% at the end of 15 h. Yet, RTX-2 formulation maintained the same integrity up to the end of 18 h. The highest swelling percentage of 135.80 ± 2.47% (RSX-1) and 163.29 ±1.22% (RSX-2) was found for the tablets prepared with thiolated and S-protected thiolated polymer, thus indicating an improvement in hydration capacity. This is attributed to the occurrence of thiol moiety on xanthan gum, which was accountable for intrachain disulfide bond formation and made the polymeric matrix, which can absorb and retain the water. Furthermore, the S-protected formulation remained stable for an extended period without erosion in contrast to residual batches. The ex vivo mucus adhesion time was estimated using a glass-slide modified dissolution apparatus, and the results were compared. The mucoadhesion property of the preparation consisting of xanthan gum may exhibit weak bonds (non-covalent type) with cysteine sub-domains of the mucus, which correspondingly showed less mucoadhesion time than the thiolated and S-protected preparations. RSX-1 and RSX-2 exhibited greater residence periods due to the formation of potent covalent bonds through disulfide–thiol interchange reactions with mucosa [Figure 7]. 

Due to thiolation, the residence time and adhesion force were raised around 2–3-fold (as shown in Table 3) [56]. Thiolated formulations require maximum force for detachment from mucus as they have strong disulfide bonds with the stable matrix. There was no erosion and breakdown during the study. The residence period and mucoadhesion potential were improved correspondingly with the concentrations of STX in RSX-1 and RSX-2 formulation because of the high affinity towards the mucus gel layer. Formulations with unmodified xanthan gum showed moderate mucoadhesion time and strength [Table 7].

### 3.4. Cell Viability Analysis by Resazurin Assay

**Figure 8:**
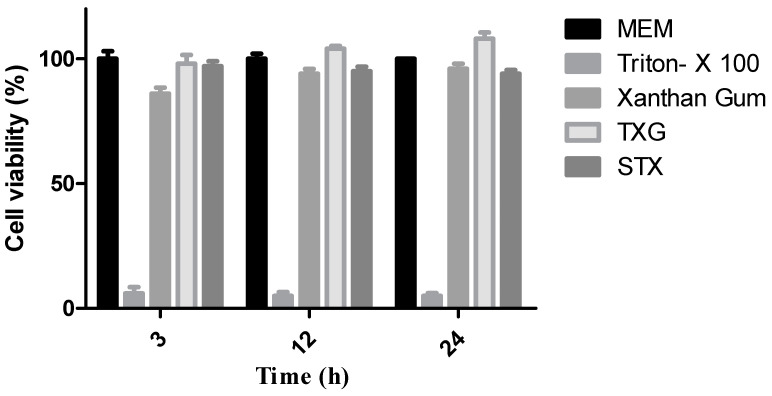
Cytotoxic studies.

Cell viability analysis of xanthan gum, TXG, and STX was conducted on the CaCO-2 cell cultures to determine cytotoxicity using a resazurin assay. Resazurin is a less fluorescent blue dye, which can be irreversibly reduced to resorufin (pink color with high fluorescence). It is extensively employed to study cytotoxic effects and determine the metabolic activity of cells by the in vitro method. Resorufin is detectable by spectrofluorimetric with an excitation and emission wavelength of 540 nm and 590 nm, respectively. Cells were exposed to the above samples for 3, 12, and 24 h. The results depicted as histograms in Figure 8 revealed that all the thiolated and S-protected polymers do not have cytotoxic effects as their cell viability was very high. In addition, there were no remarkable changes after 12 and 24 h. After 24 h, statistically essential changes were seen regarding cell viability for STX referred to as 3 h incubation. Although the polymer exhibits many covalently bound thiol moieties, there may be proximal thiol within the same chain, which can react quickly among themselves instead of the thiols present on other polymer chains. This could cause greater viscoelastic characters of thiomers and their decreased cell viability. Hence, the S-protected polymers are regarded as safe for in vivo usage.

### 3.5. In Vitro Drug Release Studies

**Figure 9:**
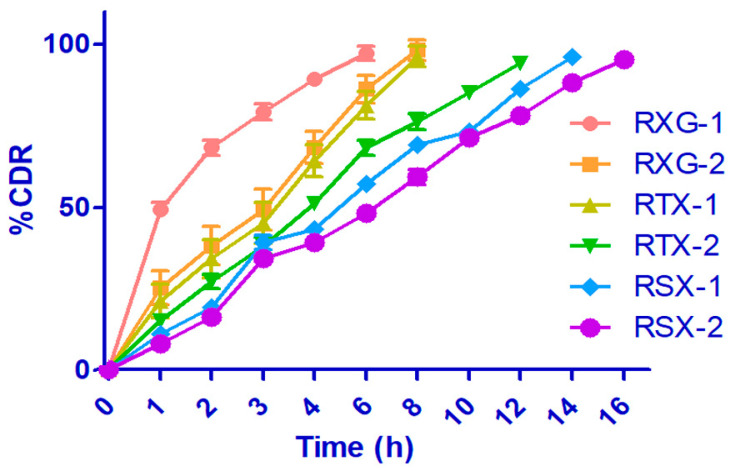
In vitro drug release profile of repaglinide mucoadhesive formulations. (CDR—cumulative drug release).

The dissolution test was conducted using the in vitro modified basket method to imitate the in vivo mucoadhesion of the drug system. Figure 9 shows the dissolution profile of all the formulated preparations. The unmodified OG tablets showed 98.97 and 99.68% drug release by the last 7 and 9 h. Initially, RGM-1 and RGM-2 controlled the drug release until 4 h because of quick hydration and swelling. However, they could not continue the same integrity of the tablet matrix and thus the burst effect, causing drug release. These findings were noted to agree with the previous ex vivo mucoadhesion time. The preparations having 25 and 40% SA exhibited controlled release until the last 10 and 13 h, respectively. Drug release deceleration increased (RGM-5,16 h; RGM-6,19 h) for the preparations with TOG. Thiolation renders information of 3D gel organization and inter-/intrachain disulfide bonds (this could enhance the cross-linkage and cohesive nature of the matrix), therefore improving the passage for the media diffusion. Drug release kinetics of RSX-2 follows controlled release with anomalous (non-Fickian) diffusion mechanism (slop value of Korsmeyer–Peppas model- n = 0.9354). 

### 3.6. Pharmacokinetic Studies

**Figure 10:**
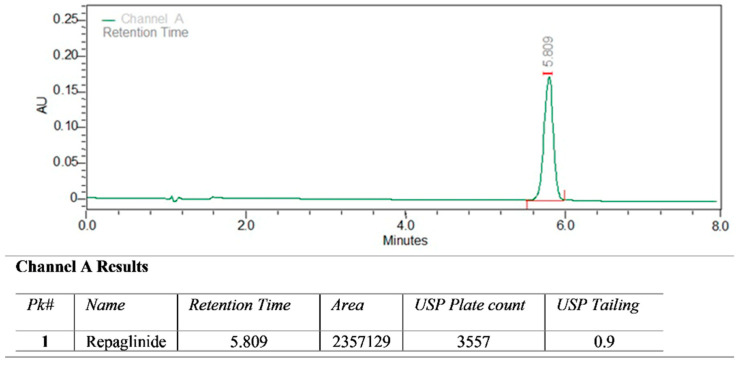
A typical chromatogram of repaglinide by RP-HPLC.

**Figure 11:**
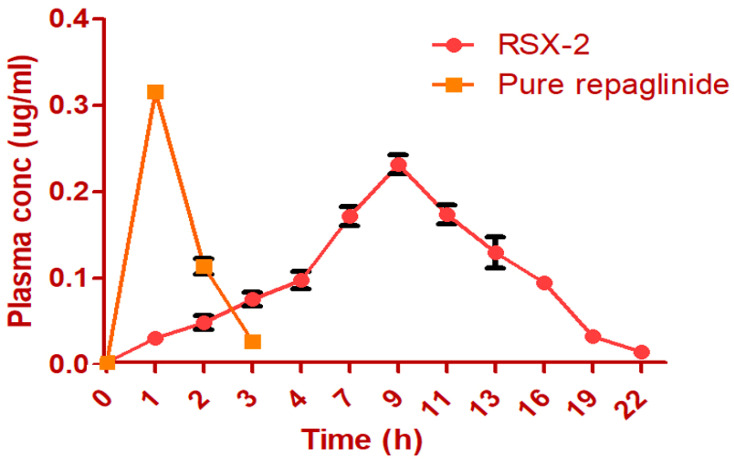
Plasma concentration–time profile for RSX-2 and pure repaglinide (n = 6).

Repaglinide was studied using the RP-HPLC technique. There was no interference of the blank solution during the retention period; this ensures the precision of the method. The retention period of drug repaglinide was observed to be 5.809 min [Figure 10]. A typical chromatogram of repaglinide was observed at 245 nm. Various PK parameters for the pure-repaglinide solution and RSX-2 were computed as mentioned in Table 8. This data of RSX-2 and pure drug certainly represent those peak concentrations (Cmax) of repaglinide which were attained quickly and eliminated more quickly, in contrast to the S-protected thiolated preparation. The AUC for RSX-2 was improved more than 50 times, denoting a better absorption and enhanced relative bioavailability [Table 8]. This may be because of high thiomers concentration. K_a_ for RSX-2 was decreased to 0.252 (1/h), and elimination t_1/2_ was 4.587 h, which is preferable for controlled drug forms [Figure 11]. V_d_ is nearly the same for both samples. The improved gastric retention of thiolated preparations enhances the time required for dissolving the drug repaglinide prior to invading the intestines, facilitating an improved bioavailability.

## 4. Conclusions

Thiomers have the potential to form inter- and/or intramolecular disulfide bonds which render an improvement in swelling nature, mucoadhesion potential, and sustained-release nature. The thiolation of xanthan gum was optimized by response surface methodology along with statistical analysis. Thiolation was carried out by esterifying xanthan gum with TGA resulting in conjugates with more enhanced properties than the original material. The enhanced mucoadhesion potential was observed on the S-Protection of thiolated xanthan gum. STX was synthesized and characterized for rheological properties and the quantitative analysis of thiol/sulfide/MA groups. Repaglinide mucoadhesive tablets were formulated using two different concentrations of xanthan gum, TXG and STX. Furthermore, the thiolated tablets remained stable for an extended time duration without disintegration, in contrast to residual batches. Preparations with SA showed a modest mucoadhesion time and strength. Synthesized S-protected polymer can be regarded as safe for in vivo use. The cell viability test by resazurin assay confirmed the safety of the novel thiolated and S-protected thiolated polymers. In vitro release and drug release studies improved the passage for the media diffusion to release repaglinide in a controlled manner. The kinetic studies of RSX-2 indicated a decrease in the K_a_ and K_e_, which is favorable for control release. 

## Figures and Tables

**Table 1:** Experimental plan for Box–Behnken design in terms of actual and coded values.

Factors/Independent Variables	Levels					Responses/Dependent Variables	Constraints
	−1.414	−1	0	+1	+1.414		
TGA Conc- X_1_ (moles/L)	1.17	2	4	6	6.82	Viscosity (mPa-mPs)	Maximum
Xanthan Gum Conc-X_2_ (g/L)	3.17	4	6	8	8.82	MS (h)	Maximum

**Table 2:** Formulation of repaglinide gastro retentive mucoadheisve tablets *.

	RXG-1	RXG-2	RTX-1	RTX-2	RSX-1	RSX-2
Xanthan gum (%)	30	60	--	--	--	--
TXG (%)	--	--	30	60	--	--
STX (%)	--	--	--	--	30	60

* Every formulation consists of repaglinide (10 mg), Magnesium stearate (4 mg), talc (4 mg) and microcrystalline cellulose (MCC)—an adequate amount to produce 150 mg tablet.

**Table 3:** Experimental runs projected and their responses observed.

		Factor 1	Factor 2	Response 1	Response 2
Std	Run	A:TGA Conc	B:Xanthan Gum Conc	Viscosity	MS
		(moles/L)	(g/L)	mPa-s	h
1	13	2	4	15.5	2.5
3	1	2	8	17.5	2.9
5	10	1.17157	6	18.7	1.5
7	12	4	3.17157	20.4	3.8
8	9	4	8.82843	22.4	5.4
13	2	4	6	27.4	8.4
10	8	4	6	27.5	8.5
11	11	4	6	27.5	8.2
9	3	4	6	27.9	8.3
12	6	4	6	28.1	8.4
2	4	6	4	28.4	6.2
4	5	6	8	28.4	5.7
6	7	6.82843	6	34.5	4.6

**Table 4:** Model statistical summary.

Response	Models	R^2^	Adju.R^2^	Pred.R^2^	Adequate Precision	Sequential *p*-Value	Remarks
Viscosity	Linear	0.7398	0.6878	0.5335	----	0.0012	
	2 FI	0.7426	0.6568	0.3078	----	0.7636	
	Quadratic	0.9848	0.9740	0.8976	29.2120	<0.0001	Suggested
	Cubic	0.9858	0.9659	0.1529	---	0.8488	Aliased
MS	Linear	0.2017	0.0421	−0.2903	---	0.3242	
	2 FI	0.2044	−0.0608	−0.6586	---	0.8663	
	Quadratic	0.9763	0.9593	0.8351	20.2198	<0.0001	Suggested
	Cubic	0.9928	0.9826	0.5796	---	0.0514	

**Table 5:** Analysis of variance (ANOVA) results.

	Intercept	A	B	AB	A^2^	B^2^
Viscosity	27.68	5.76807	0.603553	−0.5	−0.9275	−3.5275
*p*-values		<0.0001	0.0061	0.2974	0.0283	<0.0001
MS	8.36	1.36051	0.270343	−0.225	−2.53	−1.755
*p*-values		0.0001	0.0017	0.4055	<0.0001	<0.0001

**Table 6:** The quantity of thiol and disulfide groups was estimated by quantitative assay.

Sample	-SH	-S-S-	MNA
		(µmol/g)	
TXG	266.78 ± 13	202.29 ± 14	--
STX	--	--	219.48 ± 11.8

**Table 7:** Comparison of ex vivo mucoadhesion time and mucoadhesion strength.

	Ex Vivo Residence Time		Mucoadhesion Strength
	Glass Slide Method	Modified Basket Method	(g)
RXG-1	410 min	385 min	1.68 ± 0.85
RXG-2	460 min	442 min	2.45 ± 1.12
RTX-1	575 min	559 min	3.89 ± 1.35
RTX-2	640 min	635 min	6.13 ± 2.04
RSX-1	>16 h	>16 h	12.78 ± 1.45
RSX-2	>16 h	>16 h	17.57 ± 1.28

**Table 8:** Pharmacokinetic parameters for repaglinide solution and test (RSX-2) preparations.

Pharmacokinetic Parameter	Pure Repaglinide	RSX-2
C_max_ (μg mL^−1^ h)	0.313 ± 0.020	0.229 ± 0.015
t _max_ (h)	1 ± 0.000	9.000 ± 0.000
AUC_0-t_ (μg mL^−1^ h)	0.395 ± 0.251	2.865 ± 0.084
AUMC_0-t_ (μg mL^−1^ h)	0.49 ± 0.321	25.481 ± 1.249
MRT_t_ (h)	1.25 ± 0.268	10.746 ± 0.142
t_1/2_ (h)	0.41 ± 0.084	4.587 ± 0.247
AUC_0-i_ (μg mL^−1^ h)	0.396 ± 0.0.324	2.905 ± 0.128
AUMC_0-i_ (μg mL^−1^ h)	0.496 ± 0.415	27.645 ± 1.089
CL (l/h)	2.52 ± 0.159	0.801 ± 0.031
V_d_ (l)	1.48 ± 0.098	2.948 ± 0.391
K_e_ (1/h)	1.690	0.252

## Data Availability

Not applicable.

## Supplementary Materials

The following supporting information can be refred at the template: https://www.mdpi.com/article/10.3390/polym14173529/s1, Figure S1: Normal probability and residuals plots for (a) viscosity and (b) MS.
