# Supplementary material for: Development of Novel S-Protective Thiolated-Based Mucoadhesive Tablets for Repaglinide: Pharmacokinetic Study
Source: Polymers (Basel). 2022 Aug 28;14(17):3529. doi: 10.3390/polym14173529 (PMC9460926; doi:10.3390/polym14173529)
Supplement: Supplementary file 1 [file polymers-14-03529-s001.zip › polymers-1846889-supplementary.pdf]

Supplementary

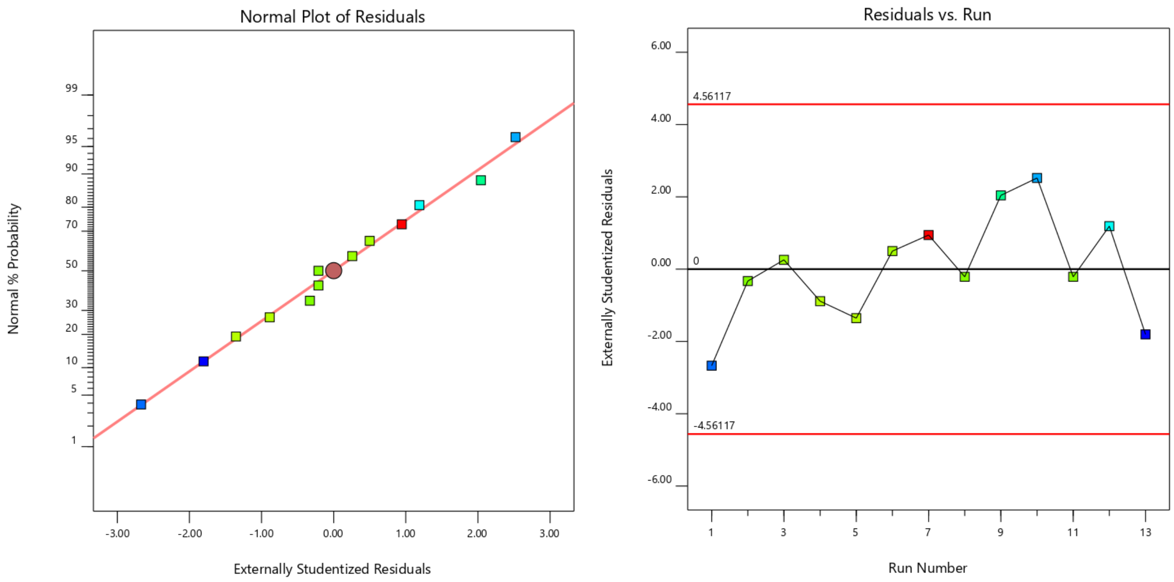

(a)

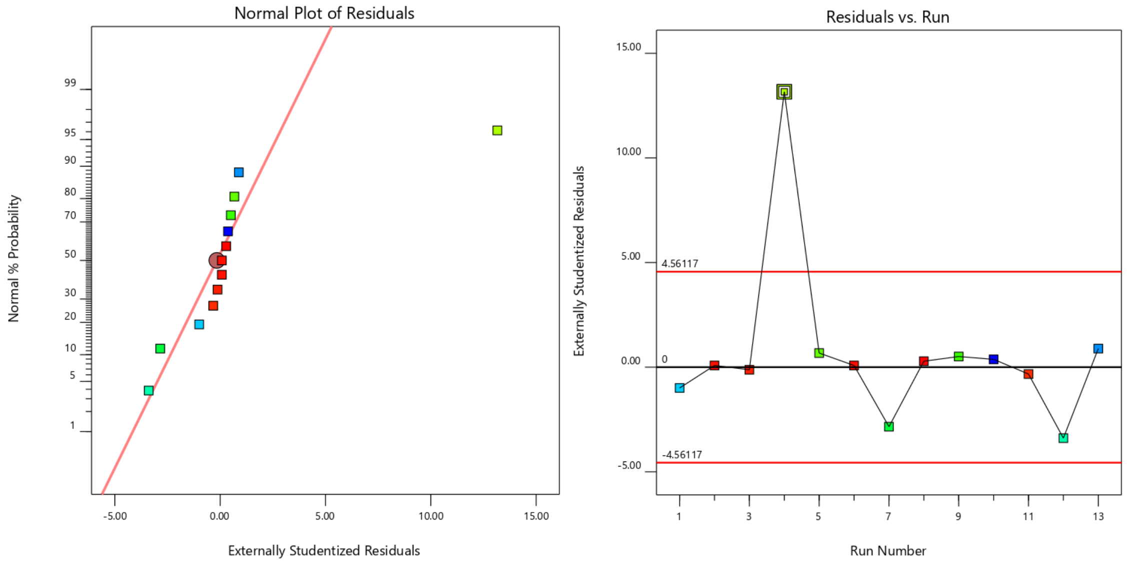

(b)

Supplementary Figure S1. Normal probability and residuals plots for (**a**) viscosity and (**b**) MS
